# Presence of gustatory and olfactory dysfunction in the time of the COVID-19 pandemic

**DOI:** 10.1186/s12879-021-06294-2

**Published:** 2021-06-26

**Authors:** Alexander Kusnik, Christel Weiss, Melanie Neubauer, Bianca Huber, Marlis Gerigk, Thomas Miethke, Nicole Hunter, Nicole Rotter, Sonja Ludwig, Angela Schell, Matthias P. Ebert, Andreas Teufel

**Affiliations:** 1 Department of Medicine II, University Medical Center Mannheim, Medical Faculty Mannheim, Heidelberg University, Mannheim, Germany; 2 Clincial Cooperation Unit Healthy Metabolism, Center for Preventive Medicine Baden-Württemberg, Medical Faculty Mannheim, Heidelberg University, Mannheim, Germany; 3 Department of Statistics, Biomathematics and Information Processing, Heinrich Lanz Center for Digital Health, Medical Faculty Mannheim, Heidelberg University, Mannheim, Germany; 4 Institute of Medical Microbiology and Hygiene, Medical Faculty of Mannheim, University of Heidelberg, Mannheim, Germany; 5 Mannheim Institute for Innate Immunoscience (MI3), Medical Faculty of Mannheim, Heidelberg University, Mannheim, Germany; 6 Department of Otorhinolaryngology, Head and Neck Surgery, Medical Faculty Mannheim, Heidelberg University, Mannheim, Germany; 7 Department of Medicine II, Division of Hepatology, Division of Clinical Bioinformatics, University Medical Center Mannheim, Medical Faculty Mannheim, Heidelberg University, Mannheim, Germany

**Keywords:** COVID, COVID-19, SARS-CoV-2, Anosmia, Smell, Hyposmia, COVID-19 negative Dysgeusia, Taste, Loss, Gustatory, Olfactory, Olfaction

## Abstract

**Background:**

The unexpected outbreak of the novel severe acute respiratory syndrome coronavirus 2 (SARS-CoV-2) caused more than 49 million cases and an estimated 2,000,000 associated deaths worldwide. In Germany, there are currently more than 2,000,000 laboratory-confirmed coronavirus disease 2019 (COVID-19) cases including 51,800 deaths. However, regional differences also became apparent and with the second wave of infections, the detailed characterization of COVID-19 patients is crucial to early diagnosis and disruption of chains of infections.

**Methods:**

Handing out detailed questionnaires to all individuals tested for COVID-19, we evaluated the clinical characteristics of negative and positive tested individuals.

Expression of symptoms, symptom duration and association between predictor variables (i.e. age, gender) and a binary outcome (olfactory and gustatory dysfunction) were assessed.

**Results:**

Overall, the most common symptoms among individuals who tested positive for SARS-CoV-2 were fatigue, headache, and cough. Olfactory and gustatory dysfunction were also reported by many SARS-CoV-2 negative individuals, more than 20% of SARS-CoV-2 negative tested individuals in our study reported olfactory and gustatory dysfunction. Independent of SARS-CoV-2 status, more females displayed symptoms of gustatory (29.8%, *p* = 0.0041) and olfactory dysfunction (22.9%, *p* = 0.0174) compared to men.

**Conclusions:**

Bringing early SARS-CoV-2 tests to the populations at risk must be a main focus for the upcoming months. The reliability of olfactory and gustatory dysfunction in COVID-19 negative tested individuals requires deeper investigation in the future.

**Supplementary Information:**

The online version contains supplementary material available at 10.1186/s12879-021-06294-2.

## Introduction

The novel COVID-19 coronavirus infection currently causing a global pandemic may present on a spectrum from asymptomatic to severe infection affecting almost every possible organ system [1]. A more severe course of disease, with rapid deterioration and acute respiratory distress syndrome (ARDS) or even fatal outcomes were reported especially among older people or those with comorbidities [2]. The current literature estimated a mean incubation period of three to 5 days [3, 4]. In symptomatic patients, the clinical manifestations of the disease usually started after less than 1 week [4]. However, as the virus effectively replicates in the upper respiratory tract and infected individuals produce a considerable amount of virus during a prodrome period, the infection may be spread early and index persons may be unrecognized for several days [2]. As a second wave of COVID-19 is ongoing, epidemiological studies are important in order to characterize symptoms, comorbidities, age and even country specific characteristics of the disease in order to identify infected patients as early as possible [4]. We therefore report our single center study on COVID-19 and share our detailed analysis on patient-reported symptoms, co-morbidities, and course of disease.

## Methods

### Subjects and setting

A questionnaire was developed according to relevant symptoms assessing general patient characteristics, severity and duration of symptoms and use of medication. It was distributed between March 31 and July 15, 2020 after COVID-19 testing to each person. Adult patients with a possible SARS-CoV-2 infection received testing via real-time reverse transcription PCR (rRT-PCR) of nasopharyngeal/oropharyngeal swabs in Mannheim, Germany. Most individuals received SARS-CoV-2 testing because of contact with people who had tested positive for SARS-CoV-2 infection, displaying symptoms likely for a SARS-CoV-2 infection, or because they had travelled in an area of high risk of exposure.

### Statistical methods

Statistical Package for the Social Sciences for Windows (SPSS version 26,0; IBM Corp, Armonk, NY, USA) and SAS, release 9.4 (SAS Institute Inc., Cary, North Carolina, USA) were used to perform the statistical analyses.

In order to compare two groups regarding a binary factor (i.e. co-morbidity present or not present) Chi2 test or Fisher’s exact test was performed, as appropriate. Expression of symptoms displayed by patients was categorized into none, mild, moderate, severe and very severe. For these ordinally scaled parameters an exact trend test according to Cochran-Armitage has been used. Quantitative variables approximately normally distributed (i.e. age) have been analyzed by a 2-sample t test. Symptom duration was evaluated with the help of a Mann-Whitney-U-Test. A multiple logistic regression was performed in order to investigate the association between predictor variables (i.e. age, gender) and a binary outcome (olfactory and gustatory dysfunction). Incomplete responses were excluded from analysis. In general, the result of a statistical test has been considered as significant for *p* < 0.05.

## Results

### Demographics and clinical characteristics

A total of 711 patients suspected of COVID-19 were included in this study, with 43 (6%) patients tested positive via PCR and 668 patients (94%) tested negative. There were 313 males and 396 females (2 unspecified). In the negative and positive tested subgroups, 44.4% or 39.5% of all individuals were male (*p* = 0.5298). The mean age of the SARS-CoV-2 negative tested subgroup was comparable with 40.9 ± 14.5 years (range 18–80) and 41 ± 16.2 years (range 20–82) in the COVID-19 positive tested subgroup (*p* = 0.9722).

### Co-morbidity

The most prevalent comorbidities of patients were lung disease (77, 10.8%), followed by other pre-existing diseases most commonly consisting of allergic diseases (34 patients 4.78%), hypertension (38 patients, 5.34%), endocrine disorders (30 patients; 4.21%) and chronic heart disease (24 patients; 3.37%) (Table 1).

**Table 1:** Presence of pre-existing diseases according to COVID-19 status (n.a. = not applicable)

Variable	COVID-19 Positive	COVID-19 Negative	***p***-value
Number of Patients	43	668	
Overall previous diseases	27 (62.79%)	156 (23.35%)	< 0.0001
Stroke	1 (2.33%)	2 (0.3%)	0.1709
Myocardial Infarction	2 (4.65%)	9 (1.35%)	0.1390
Chronic Heart Disease	1 (2.33%)	23 (3.4%)	1.0000
Inflammatory Bowel Disease	0	8 (1.2%)	1.0000
Rheumatological Disorder	1 (2.33%)	14 (2.10%)	0.6115
Transplantation	0	3 (0.45%)	1.0000
Kidney Disease	4 (9.30%)	3 (0.45%)	0.0004
Dialysis	0	0	n.a.
Autoimmune Liver Disease	0	1 (0.15%)	1.0000
Liver Cirrhosis	1 (2.33%)	2 (0.30%)	0.1709
Lung Disease	5 (11.63%)	72 (10.78%)	0.8010
Lupus	0	2 (0.30%)	1.0000
Hypertension	4 (9.30%)	34 (5.1%)	0.2787
Endocrine disorder	2 (4.65%)	28 (4.2%)	0.7017
Diabetes	1 (2.33%)	21 (3.1%)	1.0000
Allergic disorders	1 (2.33%)	33 (4.9%)	0.7146
Other	4 (9.30%)	69 (10.3%)	1.0000

Statistical difference in symptoms was only seen for self-reported kidney disease (*p* = 0.0004), indicating that co-morbidities were distributed similarly in positive and negative tested patients. However, among positive tested patients 63% self-reported pre-existing conditions, which was significantly higher than in the negative tested population.

### Clinical presentation

Among all individuals tested, the most commonly observed symptoms were fatigue and headache (Table 2). However significant differences were observed between COVID-19 positive and negative patients. Most common symptoms among individuals tested positive for COVID-19 were fatigue (91%), headache (79%) and cough (74%). These symptoms were also the most common symptoms displayed in COVID-19 negative patients (fatigue (70%), headache (63%) and cough (57%). Trend tests revealed statistical significance in symptoms between SARS-CoV-2 positive and negative tested individuals for fever, olfactory and gustatory disturbance and fatigue (each *p* <  0.0001), headache (*p* = 0.0073) and cough (*p* = 0.0004). In addition, statistical significance was seen for dyspnea (*p* = 0.0254), joint pain (*p* = 0.0015) and rhinorrhea (*p* = 0.0197).

**Table 2:** Presence of symptoms according to COVID-19 status

Variable	COVID-19 Positive	COVID-19 Negative	***p***-value
Number of Patients	43	668	
Fever (> 37.5 °C)	22 (51.16%)	214 32.04%)	< 0.0001
Gustatory Dysfunction	27 (62.79%)	156 (23.35%)	< 0.0001
Olfactory Dysfunction	25 (58.14%)	133 (19.91%)	< 0.0001
Visual Dysfunction	5 (11.63%)	80 (11.98%)	0.8454
Auditory Dysfunction	2 (4.65%)	55 (8.23%)	0.4569
Sensibility Dysfunction	11 (25.58%)	87 (13.02%)	0.1353
Dyspnoea	21 (48.84%)	267 (39.97%)	0.0254
Fatigue	39 (90.70%)	466 (69.76%)	0.0001
Headache	34 (79.07%)	419 (62.72%)	0.0073
Joint Pain	26 (60.47%)	309 (46.26%)	0.0015
Rhinorrhea	28 (52.69%)	352 (52.69%)	0.0197
Cough	32 (74.42%)	378 (56.59%)	0.0004
Pharyngitis	26 (60.47%)	360 (53.89%)	0.0981
Diarrhea	14 (32.56%)	177 (26.50%)	0.8507
Ocular Pruritus	18 41.86%)	211 (31.59%)	0.4021
Scrotal Pain	0	13 (4.39%)	0.7031

Individuals with the relevant symptoms who tested positive for COVID-19 reported an overall longer median duration of symptoms compared to individuals tested negative for SARS-CoV-2 (Table 3). Statistical significance in the duration of symptoms between SARS-CoV-2 positive and negative tested individuals was seen for fever (3.5 days; *p* = 0.0004), olfactory disturbance (6 days; *p* = 0.0010), dyspnea (5.5 days; *p* = 0.0355), fatigue (5 days; p = 0.0010) and pharyngitis (3,5 days; *p* = 0.0411).

**Table 3:** Median duration of symptoms based on COVID-19 status (sample sizes in parentheses)

Duration in days			
Symptom	Symptoms in COVID-19 positive patients (days)	Symptoms in COVID-19 negative patients (days)	***p***-value
Fever (> 37.5 °C)	3.5 (*n* = 22)	2 (*n* = 214)	0.0004
Olfactory Dysfunction	6 (*n* = 25)	3 (*n* = 133)	0.0010
Visual Dysfunction	2 (*n* = 5)	2.5 (*n* = 80)	0.6739
Auditory Dysfunction	1 (n = 2)	3 (*n* = 55)	0.1734
Sensibility Dysfunction	2 (*n* = 11)	2 (*n* = 87)	0.1670
Dyspnoea	5.5 (n = 21)	3 (*n* = 267)	0.0355
Fatigue	5 (*n* = 39)	3 (*n* = 466)	0.0010
Headache	3 (*n* = 34)	3 (*n* = 419)	0.0567
Cough	4.5 (*n* = 32)	3 (*n* = 378)	0.1737
Pharyngitis	3.5 (n = 26)	3 (*n* = 360)	0.0411
Diarrhea	2 (*n* = 14)	2 (*n* = 177)	0.4913

### Olfactory and gustatory dysfunction symptoms

Our questionnaire included a more detailed characterization regarding duration, onset and specifics of taste and olfactory disturbance (OGD). 62.8 and 58.1% of patients who were tested positive for SARS-CoV-2 reported gustatory or olfactory disturbance, respectively. The majority of SARS-CoV-2 positive patients with a gustatory disturbance described an overall decline in taste (46.5% *p* = 0.0001) compared to 14.22% seen in SARS-CoV-2 negative tested patients with gustatory dysfunction (Table 4). In contrast, 23.35 and 19.91% of SARS-CoV-2 negative tested patients reported gustatory and olfactory dysfunction, respectively (Table 2). 27.5% of patients who tested negative for SARS-CoV-2 reported OGD on a spectrum from mild to very severe (51 patients (7.6%) only gustatory dysfunction, 28 patients (4.2%) only olfactory dysfunction, 105 (15.7%) with the presence of both, olfactory and gustatory dysfunction).

**Table 4:** Specific description of Taste disturbance

Variable	COVID-19 Positive	COVID-19 Negative	***p***-value
General disturbance in taste	27 (62.79%)	149 (22.31%)	0.0001
No taste of sweet	3 (6.98%)	18 (2.69%)	0.1280
No taste of sour	3 (6.98%)	15 (2.25%)	0.0890
Only bitter taste	2 (4.65%)	12 (1.80%)	0.2054
Taste diminished	20 (46.51%)	95 (14.22%)	0.0001
Metallic taste	2 (4.65%)	34 (5.09%)	1.0000
Other taste disturbance	4 (9.30%)	18 (2.69%)	0.0382
Mean number of changes in taste	0.8	0.3	0.0001

We also assessed the concomitant use of anti-inflammatory medication (Table 5) in SARS-CoV-2 negative and positive patients. The analysis indicated that 60% of SARS-CoV-2 positive tested patients and 37% of SARS-CoV-2 negative tested patients used anti-inflammatory medications (*p* = 0.0019). Ibuprofen (25.6%) and acetaminophen (30.2%) were the most commonly used medications in patients suffering from COVID-19. Similar findings were seen in the SARS-CoV-2 negative tested subgroup; the most commonly used medications were ibuprofen (20.5%) and acetaminophen (13.5%). Chemosensory complaints from drugs might present differently and might include altered sensation as bitter or metallic taste and perceptual distortions [5]. We therefore assessed for more specific taste disturbances like bitter, sour and metallic taste. A statistical significance in the mean number of taste disturbances was evident in the SARS-CoV-2 positive tested subgroup (μ in changes of taste 0.8 versus 0.3; *p* <  0.0001) (Table 4). Furthermore, we analyzed for possible other related symptoms compatible with allergies (e.g. ocular pruritus, rhinorrhea) as these conditions tend to be highly underdiagnosed [6]. Patients tested negative for SARS-CoV-2 who displayed OGD more commonly reported rhinorrhea (72%, *p* = 0.0001) and ocular pruritus (39%, *p* = 0.0164) compared to negative tested patients without OGD (p = 0.0001) (Table 6). A statistical significance in the number of pre-existing diseases was evident in COVID-19 negative tested patients with OGD (59.2%, *p* = 0.0314), but pre-existent diseases possibly responsible for OGD like rheumatological disorders [7], stroke [8, 9] or kidney disease [9] were similarly distributed between both groups.

**Table 5:** Usage of medication based on COVID-19 status

Variable	COVID-19 Positive	COVID-19 Negative	***p***-value
Use of anti-inflammatory medication	26 (60.4%)	245 (36.68%)	0.0019
Ibuprofen	11 (25.6%)	137 (20.5%)	0.4271
Acetaminophen	13 (30.2%)	90 (13.5%)	0.0025
Aspirin	1 (2.3%)	25 (3.7%)	1.0000
Metamizole	6 (14.0%)	21 (3.1%)	0.0039
Diclofenac	1 (2.3%)	1 (0.1%)	0.1174
Other medication	2 (4.7%)	29 (4.3%)	0.0027

**Table 6:** Presence of symptoms in COVID-19 negative tested patients with olfactory and gustatory dysfunction (binary scaled)

Variable	COVID-19 Negative with OGD	COVID-19 Negative without OGD	***p***-value
Number of Patients	184	484	
Fever (37.5 °C)	80 (43.48%)	134 (27.69%)	0.0104
Visual Dysfunction	39 (21.20%)	41 (8.47%)	0.0001
Auditory Dysfunction	37 (20.11%)	18 (3.72%)	0.0001
Sensibility Dysfunction	38 (20.65%)	49 (10.12%)	0.0001
Dyspnoea	114 (61.96%)	153 (31.61%)	0.0001
Fatigue	165 (89.67%)	301 (62.19%)	0.0001
Headache	153 (83.15%)	266 (54.96%)	0.0001
Joint Pain	132 (71.74%)	177 (36.57%)	0.0001
Rhinorrhea	132 (71.74%)	220 (45.45%)	0.0001
Cough	139 (75.54%)	239 (49.38%)	0.0001
Pharyngitis	140 (76.09%)	220 (45.45%)	0.0001
Diarrhea	77 (41.85%)	100 (20.66%)	0.0001
Ocular Pruritus	71 (38.59%)	140 (28.93%)	0.0176
Medication			
General use of medication	82 (44.57%)	163 (33.68%)	0.0091
Ibuprofen	45 (24.46%)	92 (19.01%)	0.1192
Acetaminophen	31 (16.85%)	59 (12.19%)	0.1152
Aspirin	9 (4.89%)	16 (3.31%)	0.3348
Metamizole	12 (6.5%)	9 (1.86%)	0.0020
Diclofenac	1 (0.54%)	0	0.2754
Ketoprofen	1 (0.54%)	0	0.2754
Other medication	11 (5.98%)	18 (3.72%)	0.2005
Pre-existent diseases in COVID-19 negative tested patients with olfactory and gustatory dysfunction (binary scaled)			
Pre-existent disease	109 (59.24%)	322 (66.53%)	0.0314
Stroke	0	2 (0.41%)	1.0000
Myocardial Infarction	4 (2.17%)	5 (1.03%)	0.2690
Chronic heart disease	8 (4.35%)	15 (3.10%)	0.4291
Rheumatological disease	7 (3.80%)	7 (1.45%)	0.0706
Transplantation	0	3 (0.62%)	0.5651
Kidney disease	2 (1.09%)	1 (0.21%)	0.1854
Dialysis	0	0	n.a
Autoimmune liver disease	0	1 (0.21%)	1.0000
Liver cirrhosis	1 (0.54%)	1 (0.21%)	0.4753
Lung disease	23 (12.50%)	49 (10.12%)	0.3763
Lupus	0	2 (0.41%)	1.0000
other	52 (28.26%)	108 (22.31%)	0.1077

Further analysis indicated that SARS-CoV-2 negative tested patients with OGD more commonly described other neurological dysfunction like visual (21%, *p* = 0.0001), auditory (20%, p = 0.0001), or sensibility (21%, *p* = 0.0003) disturbance compared to SARS-CoV-2 negative tested patients without OGD, raising the question for the possible mechanism of increased neurological dysfunction without evidence of viral entry. Concerning gender and age, analysis showed that independent of SARS-CoV-2 status, more females displayed symptoms of gustatory dysfunction (29.8%, *p* = 0.0041) and olfactory dysfunction (22.9%, *p* = 0.0174) compared to males. Additionally, the simultaneous occurrence of OGD was more prevalent in females (33.08%) than males (23.96%) with statistical significance in younger patients (Suppl. Table 1). The results of the logistic regression analysis showed that gustatory dysfunction is highly associated with gender (*p* = 0.0047), with females being more affected than men (Odds Ratio = 1.659) whereas patient’s age does not play an important role (*p* = 0.1578, Odds Ratio = 0.991). In addition, occurrence of both, olfactory and gustatory dysfunction, is highly dependent on gender (*p* = 0.0105, Odds Ratio = 1.549) and age (*p* = 0.0361, Odds Ratio = 0.988 per year), with especially young females being affected the most.

## Discussion

Current evidence indicates that the clinical features of olfactory and gustatory dysfunction (OGD) might be far more prevalent in the European population than in the Asian population [10, 11], raising questions for possible new mutations [12], different expression of the angiotensin-converting enzyme 2 (ACE2)-entry receptor in different populations and different organs [13–15]. Neurons and glial cells express the ACE2 receptor offering a binding spot for the virus and subsequent neurological, olfactory, and gustatory dysfunction [16].

Niklassen et al. [17] assessed OGD in a total of 111 COVID-19 positive tested individuals at three different time intervals with the help of Sniffin’ Sticks and taste sprays/strips. The study indicated that during the acute infection with SARS-CoV-2, 21% of patients displayed anosmia and 49% hyposmia, in contrast 26% showed various degrees of gustatory dysfunction. The same tests were used in a study conducted by Huart et al. [18] distinguishing possible differences in the pathophysiology of OGD in acute cold and COVID-19 patients. A comparison of OGD was made between COVID-19 infected individuals, acute cold patients in the pre- COVID-19 era, and healthy controls. Similarly, like in the study conducted by Niklassen et al. [17], Huart et al. [18] showed that patients infected with COVID-19 have worse global, sweet and bitter gustatory scores (*p* = 0,0015; p = 0,026 and p = 0,001), suggesting a possible involvement of central olfactory structures and therefore a neuroinvasive nature of SARS-CoV-2. However, a definitive generalization of these results is due to a small cohort not possible.

Although the beforementioned studies stipulate a principal link between COVID-19 and OGD, no study can reliably compare the degree of existing OGD in patients before and after a SARS-CoV-2 infection. A high prevalence of OGD was also found in diverse studies [11, 19, 20], e.g., a multicenter European study [21] in which almost 90% of COVID-19 patients reported olfactory and gustatory dysfunction. Lechien et al. [21] assessed the impact of COVID-19 on olfactory and gustatory dysfunction by using a short version of the Questionnaire of Olfactory Disorders-Negative Statements (sQOD-NS) and the smell and taste component of the National Health and Nutrition Examination Survey. Similarly, Luers et al. [22] used a standardized 2-section questionnaire consisting of demographic data and the total nasal symptom score (TNSS) evaluating for nasal congestion, sneezing, nasal itching, and rhinorrhea. The study indicated a significant relationship between the presence of reduced olfaction and a reduced sense of taste (*P* < .001). Furthermore, recent studies suggested that the presence of self-reported olfactory and gustatory dysfunction had a high specificity as a screening criterion for COVID-19 [23] (98.7, 95% CI 97.6–99.4%) and correlated with a milder course of infection [24]. Similarly, our study indicated a significantly higher prevalence of olfactory (58.1%) and gustatory dysfunction (62.8%) (each *p* <  0.0001) and significantly longer median duration of olfactory disturbance (*p* = 0.0010) compared to SARS-CoV-2 negative tested patients.

Nevertheless, more than 20% of SARS-CoV-2 negative tested individuals in our study reported OGD. This may be critical to proceedings in diagnosis and treatment of COVID-19 as currently no recommendation on how to proceed with SARS-CoV-2 negative tested individuals is available. Although robust data are lacking one could assume that these individuals displaying severe olfactory and gustatory dysfunction as loss of smell and taste may have a higher chance of false negative tests as OGD was reported to serve as a potential predictor of infection [25–28].

In light of the increased awareness of OGD as a symptom of COVID-19 created by mainstream media, potential contributory factors like other diseases, medication- or allergies have to be taken into closer consideration. In addition, the general prevalence of olfactory and gustatory impairment is not well established.

Medications like acetaminophen and ibuprofen can cause olfactory and gustatory dysfunction although these side effects are reported rarely [29] and are especially more prevalent in older patients in conjunction with the use of other medication for chronic conditions [30]. Other viral infections might also be associated with olfactory and gustatory dysfunction [31]. The influenza virus is known to cause hyposmia and hypogeusia [32], but recent epidemiological data from Germany indicates that the influenza virus was virtually non-existent in April and subsequent months [33]. Additionally, we included questions assessing concomitant use of anti-inflammatory medication as the onset of taste or smell dysfunction could coincide with the introduction of a new drug or drug combination [5]. Patients infected with COVID-19 tended to use more anti-inflammatory medication compared to COVID-19 negative tested individuals with statistical difference in the use of acetaminophen (*p* = 0.0025) and metamizole (*p* = 0.0039). Therefore, our study indicates that use of concomitant medication may have a contributory effect in patients tested positive for SARS-CoV-2 reporting OGD, as a statistical significance in the mean number of taste disturbances was evident in the SARS-CoV-2 positive tested subgroup (μ in changes of taste 0.8 versus 0.3; *p* <  0.0001). In addition, pre-existing olfactory and gustatory dysfunction are possibly more likely in older male patients [5, 30], in patients with upper airway inflammation (allergic rhinitis, rhinosinusitis) [34] or in patients with neurodegenerative disorders [35]. Our analysis indicated that OGD was more commonly recognized as a symptom in the younger population. More females displayed symptoms of gustatory dysfunction (29.8%, *p* = 0.0041) and olfactory dysfunction (22.9%, *p* = 0.0174) compared to man. Additionally, the simultaneous occurrence of OGD was more prevalent in females (33.08%) than males (23.96%) with statistical significance in younger patients.

Our data indicates that a statistical difference in the number of pre-existing diseases was present in COVID-19 negative tested patients with OGD (p = 0. 0314). Similarly, COVID-19 positive tested patients had more pre-existent diseases with statistical significance in kidney disease (*p* = 0.0004,) which potentially could contribute to the presence of OGD [9, 36]. Other possible causes for altered olfaction and gustation include upper airway inflammation (e.g allergic rhinitis, chronic rhinosinusitis with or without nasal polyps) [34, 37]. Allergic rhinitis is a common, underdiagnosed disorder which can affect people of all ages and is associated with symptoms like pruritis, rhinorrhea and nasal congestion [6, 38]. Recent research indicates that over the last 20 years, a significant rise in the total number of weed pollen sensitization, especially in younger patients took place in Germany [39]. Our study indicates that patients tested negative for COVID-19 who displayed OGD more commonly reported allergy-like features like rhinorrhea (72%, *p* = 0.0001) and ocular pruritus (39%, *p* = 0.0164) compared to negative tested patients without OGD (p = 0.0001), indicating a possible allergic component which could coincide with the COVID-19 pandemic.

We acknowledge several limitations to our study. First, our study contains a relatively small amount of COVID-19 positive tested individuals. Furthermore, no psychophysical evaluation of smell and taste was conducted. Recent studies [40] indicate that an olfactory disorder in COVID-19 patients is much more prevalent than those detected by questionnaires and use of objective methods are useful tools to discriminate between these patients. That would imply that the self-reported OGD of more than 20% in our SARS-CoV-2 negative tested individuals are severely underestimated. So the correlation between OGD and different disease processes remains to be further enlightened. One can speculate about the different etiologies for OGD, but this study indicates that the global COVID-19 pandemic and our findings in COVID-19 negative-tested individuals merit deeper investigation in these disturbances in the future.

## Conclusion

Overall, when characterizing the course of COVID-19 disease in Germany, the most common symptoms among individuals who tested positive for COVID-19 were fatigue, headache, and cough. Additionally, olfactory and gustatory dysfunction were also reported by many COVID-19 negative individuals who more commonly reported allergy-like features like rhinorrhea (72%, *p* = 0.0001) and ocular pruritus (39%, *p* = 0.0164) indicating a possible allergic component that could coincide with the COVID-19 pandemic.

More females displayed symptoms of gustatory dysfunction (29.8%, *p* = 0.0041) and olfactory dysfunction (22.9%, *p* = 0.0174) compared to males. Thus, bringing early COVID-19 tests to the populations at risk must be a main focus for the upcoming months.

## Supplementary Information

**Additional file 1.**

## Data Availability

All data generated or analysed during this study are included in this published article [and its supplementary information files].
