# Supplementary material for: Presence of gustatory and olfactory dysfunction in the time of the COVID-19 pandemic
Source: BMC Infect Dis. 2021 Jun 26;21:612. doi: 10.1186/s12879-021-06294-2 (PMC8234756; doi:10.1186/s12879-021-06294-2)
Supplement: Supplementary file 1 — Additional file 1. [file 12879_2021_6294_MOESM1_ESM.docx]

**Presence of gustatory and olfactory dysfunction in the time of the COVID-19 pandemic**

Alexander Kusnik^1,2^, Christel Weiss^3^, Melanie Neubauer^1^, Bianca Huber^1^, Marlis Gerigk^4^, Thomas Miethke^4,5^, Nicole Hunter^4^, Nicole Rotter^6^, Sonja Ludwig^6^, Angela Schell^6^, Matthias P. Ebert^1,2^, Andreas Teufel^1,2, 7^

^1^Department of Medicine II, University Medical Center Mannheim, Medical Faculty Mannheim, Heidelberg University, Mannheim, Germany

^2^Clincial Cooperation Unit Healthy Metabolism, Center for Preventive Medicine Baden-Württemberg (CPM^BW^), Medical Faculty Mannheim, Heidelberg University, Germany

^3^Department of Statistics, Biomathematics and Information Processing, Heinrich Lanz Center for Digital Health, Medical Faculty Mannheim, Heidelberg University, Mannheim, Germany

^4^Institute of Medical Microbiology and Hygiene, Medical Faculty of Mannheim, University of Heidelberg, Germany

^5^Mannheim Institute for Innate Immunoscience (MI3), Medical Faculty of Mannheim, University of Heidelberg, Germany

^6^Department of Otorhinolaryngology, Head and Neck Surgery, Medical Faculty Mannheim, Heidelberg University, Germany

^7^Department of Medicine II, Division of Hepatology, Division of Clinical Bioinformatics, University Medical Center Mannheim, Medical Faculty Mannheim, Heidelberg University, Mannheim, Germany

Key words: COVID · COVID-19 · SARS-CoV-2 · Anosmia · Smell · Hyposmia · COVID-19 negative Dysgeusia · Taste · Loss · Gustatory · Olfactory · Olfaction

**Correspondence**

Andreas Teufel, M.D., Ph.D.

Department of Medicine II, Division of Hepatology,

Mannheim University Medical Center,

University of Heidelberg

Theodor-Kutzer-Ufer 1-3

68167 Mannheim

Germany

Tel: +49 621 383 4983

Fax: +49 621 383 1467

email: andreas.teufel@medma.uni-heidelberg.de

**Suppl. Table 1:** Presence of OGD in relation to gender and age

| **Outcome** | **Male** | **Female** | **p-value Chi^2^ test** |
| --- | --- | --- | --- |
| Gustatory Dysfunction | 63 (20.13%) | 118 (29.80%) | \| 0.0034 \| \| --- \| |
| Olfactory Dysfunction | 49 (15.65%) | 91 (22.98%) | \| 0.0150 \| \| --- \| |
| OGD together present | 75 (23.96%) | 131 (33.08%) | \| 0.0079 \| \| --- \| |

Presence of OGD in relation to age

| **Outcome** |  | **Mean Age** | **p-value t-test** |
| --- | --- | --- | --- |
| Gustatory Dysfunction |  | 39.69 ± 13.65 | \| p = 0,1342 \| \| --- \| |
| No Gustatory Dysfunction |  | 41.57 ± 14.94 | \|  \| \| --- \| |
|  |  |  |  |
| Olfactory Dysfunction |  | 39.23 ± 13.24 | \| p = 0,0929 \| \| --- \| |
| No Olfactory Dysfunction |  | 41.55 ± 14.94 | \|  \| \| --- \| |
|  |  |  |  |
| OGD together present |  | 39.23 ± 13.55 | \| p = 0,0296 \| \| --- \| |
| No OGD together present |  | 41.85 ± 15.01 | \|  \| \| --- \| |

Presence of OGD in relation to gender and age

| **Outcome** | **Gender** | **Age** |  |
| --- | --- | --- | --- |
| Gustatory Dysfunction | p = 0.0047 OR = 0.5065 | p = 0.1578 OR = 0.991 |  |
| Olfactory Dysfunction | p = 0.0162 OR = 1.604 | p = 0.0986  OR = 0.989 |  |
| OGD together present | p = 0.0105 OR = 1.549 | p = 0.0361 OR = 0.988 |  |
